# Proliferative retinopathy and neovascularization of the anterior segment in female type 2 diabetic rats

**DOI:** 10.1186/1758-5996-5-68

**Published:** 2013-11-13

**Authors:** Jorge E Mancini, Juan O Croxatto, Juan E Gallo

**Affiliations:** 1 Nanomedicine & Vision Group, Facultad de Ciencias Biomedicas, Department of Ophthalmology, Hospital Universitario Austral, Universidad Austral, Pilar, Buenos Aires, Argentina; 2 Department of Ophthalmic Pathology, Fundación Oftalmológica Argentina “Jorge Malbran”, Buenos Aires, Argentina

**Keywords:** Type 2 diabetes, Diabetic retinopathy, Diet, Neovascularization, Animal model

## Abstract

**Background:**

To examine the presence of diabetic retinopathy in a female rat model of type 2 diabetes fed on a high-fat diet (HFD).

**Methods:**

Wistar rats were injected with streptozotocin (STZ) at the age of two days and fed on an HFD from eight weeks onwards. Five diabetic animals were euthanized at 110 weeks of disease, together with a control group of age-matched, non-diabetic animals. A group of diabetic animals at 57 weeks of disease was included for comparison. Cross sections of the rats’ corneas, iris and retinas were histologically examined and analysed by immunohistochemistry and immunofluorescence, using glial-fibrillary-acidic-protein (GFAP), the vascular endothelial growth factor (VEGF) and the Von Willebrand factor (vWF). The trypsine digestive technique was used for the pericytes count.

**Results:**

Neovascularization was only found in the retinas, irises and corneas of the diabetic animals of 110 weeks of disease. There was also a significantly lower number of pericytes in these animals than in the controls.

**Conclusion:**

The female rat model of type 2 diabetes fed on an HFD may prove useful in evaluating the mechanisms involved in diabetic retinopathy, together with strategies to reduce its severity.

## Background

The prevalence of diabetes is increasing worldwide [1,2]. Factors involved in this special epidemia include a sedentary lifestyle, a fatty diet and being overweight. As a result, secondary diabetic complications have also risen [2]. Diabetic retinopathy is still the main cause of blindness in adults, despite important therapeutic advances.

The type 1 diabetes rat model has been widely used by visual scientists to analyse molecular mechanisms associated with diabetic retinopathy [3]. These animals are extremely fragile and die early, before retinal angiogenesis occurs. However proliferative retinopathy has not yet been reported in type 2 diabetic rats, who have a longer and healthier life [3-5]. This motivated investigators to seek new rat models of type 2 diabetes in which proliferative changes could be studied. It is worth mentioning that choroidal and retinal neovascularization have been described in rat models without diabetes [3,6], unveiling mechanisms similar to those reported in proliferative diabetic retinopathy [7].

In a previous study, we aimed to develop a proliferative diabetic retinopathy animal model in male type 2 diabetic rats fed on a high-fat diet (HFD) with a follow-up of 90 weeks. However, no proliferative changes were found (unpublished data). For this reason, we decided to conduct a new and longer study. This time the investigation was performed in a group of female rats, due to recent reports that higher levels of hyperglycaemia have been observed in female than male rats [8,9]. Female rats may develop much more severe diabetes [8,10]. In humans, recent investigations carried out in postmenopausal women reported an inverse relationship between serum concentrations of sex hormone binding globulin (SHBG) and the risk of clinical diabetes [11,12]. It is also known that oestrogens enhance angiogenesis under special circumstances, as in cancer diseases [13,14]. However, it should be noted that a difference in diabetes growth between male and female rats is still a controversial subject.

This paper reports the outcome of a research study carried out on female type 2 diabetic rats fed on an HFD, with a follow-up of 110 weeks (two years), that developed proliferative changes in the retina, iris and cornea. To our knowledge, this is the first report to demonstrate the presence of proliferative diabetic retinopathy and neovascularization of the anterior segment in rats.

## Methods

### Rat model of diabetes

Pregnant Wistar Rats (provided by CONEA, Buenos Aires) were housed in the animal facilities section of the School of Biomedical Sciences of Austral University, at 21 ± 1°C on a 12-h light–dark cycle. They were examined daily until delivery (at 9 am and 6 pm). Two days after birth, the newborn rats were intraperitoneally injected with streptozotocin (STZ) (45 mg/kg) (Sigma Lab), in 0.1 ml of a 0.1 M solution of citrate buffer of 154 mm of NaCl at 4.5pH [9,15,16]. The rat pups remained with the mothers until they were 21-days-old. Eight weeks later, the STZ-treated animals were fed with a home-made HFD. Table 1 shows the composition of the diet, which was prepared every week and stored at -7°C. Glycaemia levels were measured one week after starting the diet and every month thereafter. Blood samples of 32 μl were collected by tail snipping and tested using the Reflotron System Boehringer Mannheim. High levels of glycaemia (160-220 mg/dl) were found in rats fed on the HFD [17]. Two groups of twelve and five animals were euthanised at 57 and 110 weeks of diabetes, respectively.

**Table 1:** The composition of the high-fat diet (HFD) and standard diet

		**HFD**		**Standard**	
		**Total**	**Subtotal**	**Total**	**Subtotal**
Carbohydrates		28.8%		54.2%	
Protein		23.6%		23.6%	
Amino acids		2.2%		2.2%	
	Lysine		51.1%		51.1%
	Methionine		26.6%		26.6%
	Threonine		22.2%		22.2%
Fat		25.6%		2.7%	
	Saturates		46.4%		24.2%
	Monounsaturated		47.5%		30.2%
	Polyunsaturated		6.3%		45.6%
Ions	Calcium	1.6%		1.4%	
	Sodium	0.05%		0.05%	
	Magnesium	0.18%		0.18%	

### Control rats

Non-diabetic animals with no STZ injection were fed on standard food with a 2.71% fat content (Table 1). Their levels of glycaemia were normal (80-95 mg/dl). Twelve controls animals were euthanized at 62 weeks old and five at 115 weeks old.

Animals used in the study were handled according to the Association of Research in Vision and Ophthalmology (ARVO) Statement for the use of animals in ophthalmic research.

### Clinical parameters

Each animal was weighed using a standard scale at eight weeks of life and before death. Lipidaemia levels were determined using the Reflotron System every month and before the animal was euthanised.

### Fatty acid profile

The chemical compound of the HFD and conventional diets are shown in Table 1. In brief, the HFD contained 25.6% of fat (46.4% saturated, 47.5% monosaturated and 6.3% polysaturated) while the conventional diet contained 2.71% of fat (24.2% saturated, 30.2% monosaturated and 45.6% polysaturated). The diet’s chemical compound was analysed using animal and vegetable fats and oil-analysis by gas chromatography of methyl esters of fatty acids according to The International Organization for Standardization (ISO 5508: 1990-E).

### Clinical photographs and gross sections

Clinical photographs were taken of the animals when they were still alive and before anaesthesia. For the gross sections, we removed the ocular globe, then used a 30G needle to inject an air bubble through the corneal limbus into the anterior chamber to make the presence of blood vessels in the cornea more evident. Both pictures were taken with a NIKON digital camera DS3000 (Tokyo, Japan) using the macro function.

### Histological examination

The rats were anaesthetised using 350 mg/kg of chloral hydrate, delivered by an intraperitoneal injection. The eyes were removed and fixed in 4% paraformaldehyde (Sigma-Aldrich, St Louis, MO). Rats were then euthanised using an overdose of chloral hydrate. The eyes were left for one day for fixation, then immersed for cryoprotection in four concentrations of glucose (5% overnight and 7.5%, 10% and 20% for two hours each) and interlocked with resin. Ten-micron cryosections were obtained (Shandon AS325 Retraction) and stained with haematoxylin and eosin (H&E) as well as periodic acid-Schiff stain (PAS) for microscopic examination using an Eclipse Nikon E800 Microscope (Tokyo, Japan). At least five sections per eye were examined.

### Immunohistochemical and immunofluorescent analyses

The eyes were removed and fixed for 48 hours in 4% paraformaldehyde (Sigma-Aldrich, St Louis, MO). They were then immersed for cryoprotection in four concentrations of glucose (5% overnight and 7.5%, 10% and 20% for two hours each) and interlocked with resin. Ten-micron sections were obtained and fixed on polylisine-treated glass slides (Shandon AS325 Retraction).

For immunohistochemistry, the sections were first incubated in biotinylated goat anti-mouse IgG, then in an avidin-biotin peroxidase complex kit and finally in 3.3′-diaminobenzidine (DAB)/nickel solution. For immunofluorescence, axial sections were revealed using the secondary antibody goat-anti mouse with fluorescein. Immunofluorescent analysis was carried out using the Eclipse Nikon Microscope (Tokyo, Japan).

GFAP expression was analysed using the primary monoclonal antibody anti-GFAP (BIOGENEX, 4600 Norris Canyon Road, San Ramon, CA, USA).

The immunoreactivity of the vascular endothelial growth factor (VEGF) was examined using the anti-VEGF polyclonal antibody (Santa Cruz Biotechnology Inc, 2145 Delaware Avenue, Santa Cruz, CA) and the anti human-vWF (Sigma-Aldrich, St Louis, MO: f3520) was used for the Von Willebrand factor (vWF).

### Trypsin digestive technique

After the cornea was incised, the eyeball was fixed by immersing it, for a minimum of four hours, in 4% formalin buffered with 50 mM Na-K phosphate (pH 7.2). The retina was dissected and then placed in the 4% buffered formalin for a further hour. The retina was cut into a segment adequate for handling and washed overnight in running water. After that, it was incubated at 37°C in a solution of 3% trypsin (Difco 1:250) and 0.1 M tris buffer (pH 7.8) for one to three hours. The incubation was finished when the medium became cloudy and the tissue showed signs of digestion. The internal limiting membrane was peeled off in one sheet [18,19]. The network of vessels was freed of adherent retinal tissue by gentle shaking, mounted on a clean slide and allowed to dry. The preparation was stained with PAS and eosin.

### Image analysis: count of pericytes

Image analysis measurements were made using a KS400 system (Kontron Elektronic/Zeiss, Eching, Germany) and a Nikon DXM 1200 digital camera (Tokyo, Japan) mounted onto an Eclipse Nikon E-800 microscope for image acquisition. Briefly, images were digitalised in a rectangular frame of 1280 x 960 pixels using the 40X objective in the photo mode of illumination intensity. To adjust for possible defects in the illumination of the optical pathway, a low-pass image was produced for subtraction and background shading correction. After that, a grey value for image segmentation was interactively chosen. In order to define a threshold grey level, all the pixels whose grey value informative content was lower or higher than the segmentation grey were set to white and the others were set to black. The pericytes were counted in ten randomly-selected sections of each retina by one observer. The number of pericytes was normalised to the relative capillary density (number of cells per millimeter squared of capillary area – 3 pixels^2^ of capillary area = 1 mm^2^). The mean value was calculated in each animal. Samples were evaluated in a masked fashion. Statistical analysis was carried out using T-test analysis. All elements outside of the two standard deviations (SD) were eliminated.

### Iris thickness measurement

Thickness measurements of the iris were made in all Long-term diabetics animals (LT-DBT) and control animals of 110-weeks-old. We used ten samples (100x) of each iris from the pupil to the iris base, distributed between 300 μm in each sample. Samples were evaluated in a masked fashion. The image analyses were done with Image-J programme (Image Processing and Analysis in Java, NIH) and were performed using a Nikon DXM 1200 digital camera (Tokyo, Japan) mounted onto an Eclipse Nikon E-800 microscope for image acquisition. Images were digitalised in a rectangular frame of 1280 x 960 pixels using the 100x. To adjust for possible defects in the illumination of the optical pathway, a low-pass image was produced for subtraction and background shading correction. The statistical analysis was the same as we used for the pericytes.

## Results

Diabetic rats had higher glycaemia levels (range 140-416 mg/dl and mean 232 mg/dl, SD 81.9) at 110 weeks of diabetes compared to age-matched control rats (range 80-120 mg/dl and mean 100.5 mg/dl, SD 18.8). Higher levels of triglyceridaemia were observed in the diabetic animals than the controls (Table 2). The chemical composition of the diet is shown in Table 1.

**Table 2:** Weight, glycaemia, trygliceridaemia and cholesterolaemia in diabetic and control rats

**Group**	**No. rats**	**WA**	**WD**	Parameters *(SD)			
				Weight**	Gl***	Tri***	Ch***
CONTROL	12	8	--	320(30)	99.7(31.9)	N-M	N-M
DBT-HFD	12	8	0	340(28)	125(21)	N-M	N-M
CONTROL	12	62	--	524(80)	86(5.2)	91	119
DBT-HFD	12	65	57	449(76)	158(22)	114	115
CONTROL	5	115	--	266(45)	100(18.8)	113	118.5
DBT-HFD	5	118	110	279(62)	232(81)	122	115

N-M: not measured; WD: weeks on HFD; WA: weeks of age; Gl: fasting glycemia; Tri: triglyceridaemia; Ch: cholesterolaemia; * Mean (standard deviation); **gr; ***mg/dl.

Diabetic animals with fasting glycaemia < 160 mg/dl were excluded. Control animals with fasting glycaemia > 130 mg/dl were not included in the study.

**Figure 1:**
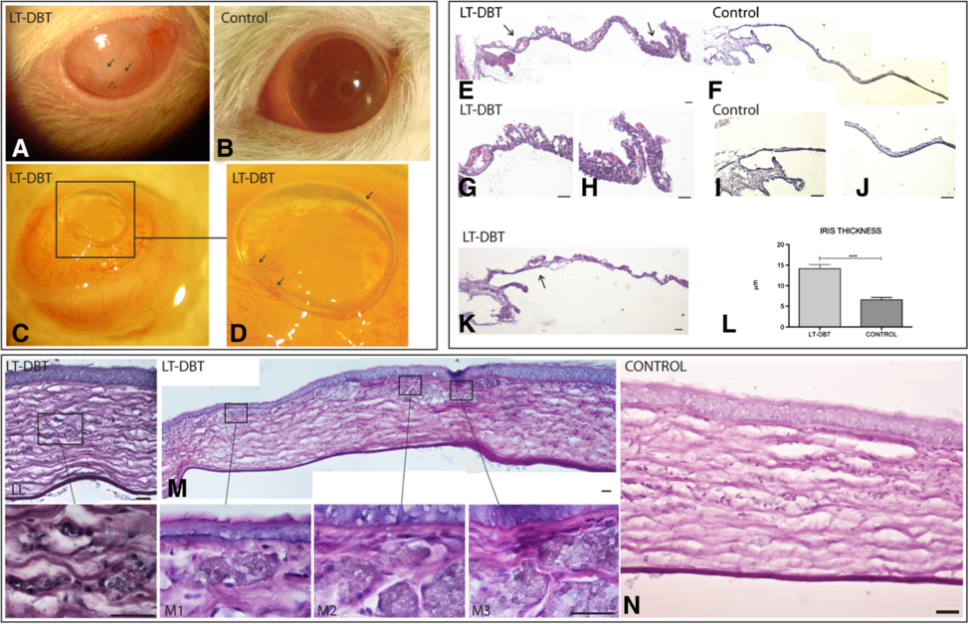
**Anterior segment vessels. A**: picture of in vivo 110-week-old animal (LT-DBT). The eye shows vessels in the cornea (arrow) and cataract (arrow head). **B**: in vivo 110-week-old control animal. **C**: gross section from an enucleated LT-DBT eye with an air bubble in the anterior chamber. **D**: Magnification from the C photograph showing the presence of vessels in the cornea (arrow). Light microscopy, PAS and eosin **(E, G, H, K)** and haematoxylin and eosin **(F, I, J)** iris staining from a LT-DBT animal **(E, G, H, K)**. Arrows indicate the presence of abnormal vessels in the iris. **F**, **I** and **J**: control iris from a non-diabetic age-matched rat. Bar 20 μm. **L**: The iris thickness is higher in the LT-DBT rats represented in μm. ***p < 0.0001, LT-DBT mean 14.11 ± 1.010 (N = 50), control mean 6.557 ± 0.5647 (N = 47). Light microscopy; haematoxylin and eosin and PAS and eosin stained corneas. LL and **M**: vessels in the corneal stroma of LT-DBT animals, LL1 and M1, 2 and 3 are magnification of vessels within the corneal stroma. **N**: normal control cornea from a LT-Control rat. Bar 20 μm.

**Figure 2:**
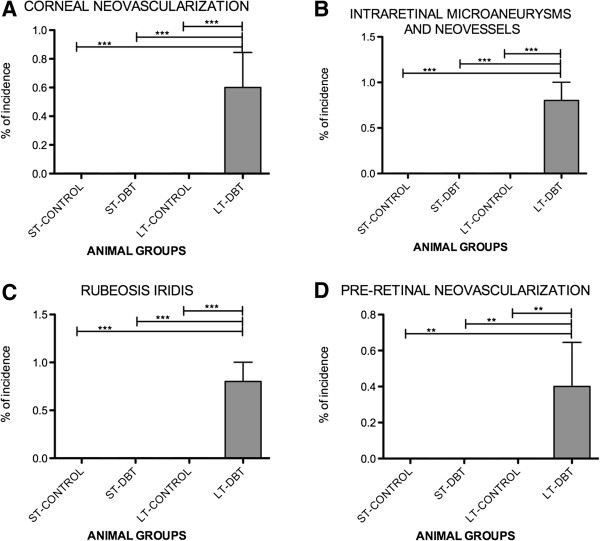
**Incidence: statistical analyses; the one-way ANOVA (and nonparametrics) was used in all the cases with the Newman-Keuls multiple comparisons post test. (A)** represents the corneal neovascularization, **(B)** intraretinal microaneurysms and neovessels, **(C)** rubeosis iridis, **(D)** presence of pre-retinal neovascularization. (p < 0,01).

When we looked at the anterior segment, the clinical photographs and gross sections showed the presence of cataracts in the five LT-DBT (110 weeks old), in two out of five 110-week-old control rats and in none of the short-term diabetic rats (ST diabetics, 57 weeks old) and 62-week-old control rats. The difference between LT-DBT rats and the other animal groups was statistically significant (p < 0.05) (Figure 1). Corneal neovascularization was seen in three out of the five LT diabetics. Neovessels were observed in the periphery and centre of the cornea, with an extension of at least one quadrant in each animal (Figure 1: A,C and D). The irises of all LT diabetic animals were significantly thicker than the controls due to the presence of abnormal vessels (Figure 1: L)(p = 0.0309, pair t-test; p < 0.0001, LT-DBT mean 14.11 μm ± 1.010 N = 50, control mean 6.557 μm ± 0.5647 N = 47). At least five neovessels were found in each iris section (Figure 1: E-K). Corneal histological analysis showed neovessels in the epithelium and anterior stroma (Figure 1: LL and M and Figure 2). No neovessels in the cornea and iris were seen in either the controls or the ST diabetics (Figure 2).

**Figure 3:**
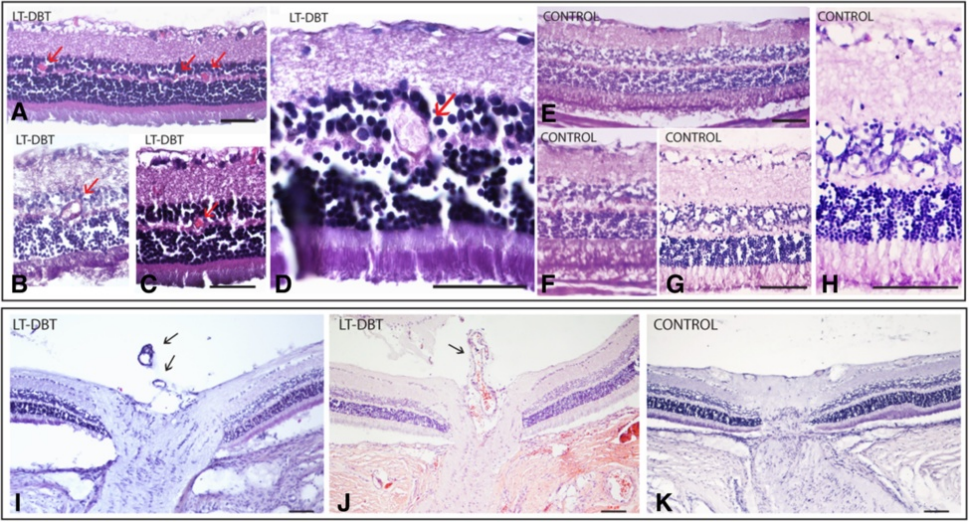
**Posterior segment.** Retina light microscopy: PAS and eosin **(A and C)** and haematoxylin and eosin **(B, D, E, F, G and H)** stained retinas. **A**, **B**, **C** and **D** are retinas from different LT-DBT animals. Arrows in picture **A**, **B**, **C** and **D** are pointing to vessel dilations in the outer plexiform layer. **E**, **F**, **G** and **H** show the LT-Control retina. Bar 20 μm. Optic nerve. Haematoxylin and eosin **(I and K)** and PAS and eosin **(J)** staining from transverse sections of the optic nerve, **I** and **J**: LT-DBT animals with vessels from nerve to vitreous (arrows) and **K**: normal optic nerve from LT-Controls. Bar 20 μm.

When we looked at the posterior segment, we saw intra-retinal neovessels in all LT diabetic animals (Figure 3). Vessel dilation or microaneurysms were found in four animals (Figure 2; Figure 3: A - D). None of these findings were seen among the controls or ST diabetics. Pre-retinal vessels were histologically seen near the optic nerve in two LT diabetics (Figure 2; Figure 3: I and J).

**Figure 4:**
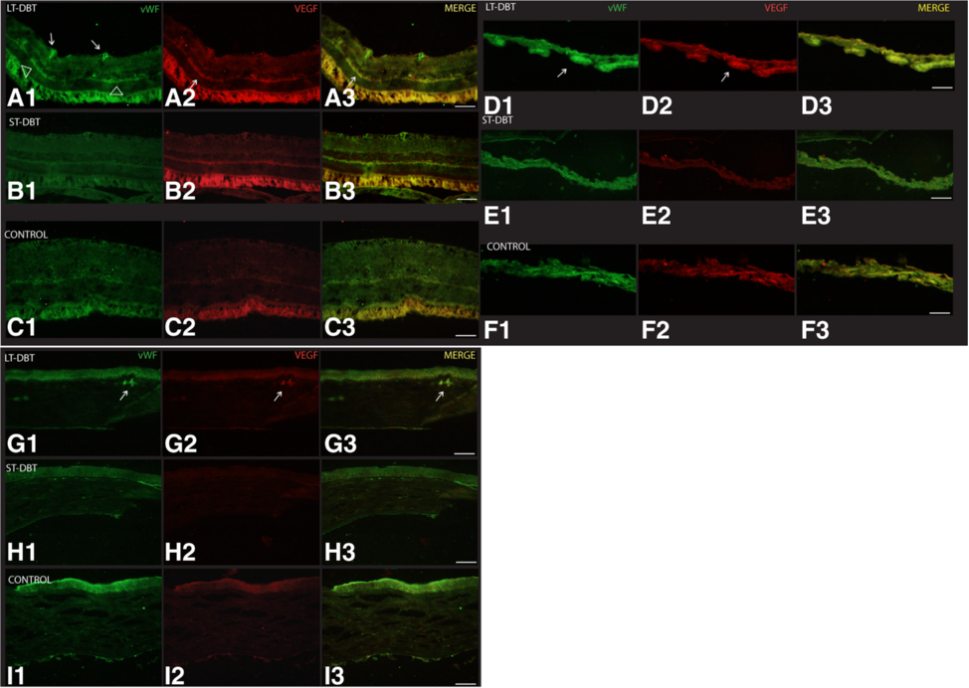
**Immunofluorescence of retina (A, B, C), iris (D, E, F) and cornea (G, H, I) from a LT-DBT animal (rows: A, D and G) and 57-week-old diabetic (rows: B, E and H) and a LT-Control animal (rows C, F and I).** Immunofluorescence of transverse retinal section for Von Willebrand antibody (vWF) (column 1 green) and VEGF (column 2 red) and merge picture (column 3 yellow). In picture **A1** the arrow points to the expression of vWF in fiber layer (FL) and arrow head in outer plexiform layer (OPL); in picture **A2** the arrow indicates an expression of VEGF in the OPL; in **A3** there is a merge picture with arrow in the OPL that indicates the co-expression of the two antibodies (vWF and VEGF). In pictures **D1**, and **D2** the arrows point to the expression of vWF and VEGF in the iris. In pictures **G1**, **G2** and **G3** the arrow indicates an abnormal expression of vWF and VEGF in the corneal stroma. 57-week-old animals. Immunofluorescent staining of transverse retinal section **(B1, B2 and B3)**, iris **(E1, E2 and E3)** and cornea **(H1, H2 and H3)** for vWF (green) and VEGF (red) and merge. At this time we found no abnormal expression of VW and VEGF. LT-Control animals. Immunofluorescent staining of transverse retinal section in picture **C1**, **C2** and **C3**: iris in **F1**, **F2** and **F3**; and cornea in **I1**, **I2** and **I3** for vWF antibody (green) and VEGF (red) and merge. No expression was found in control animals. Bar 20 μm.

The immunoreactivity for VEGF and vWF in the retina, iris and cornea was analysed by anti-vWF antibodies (Figure 4, column 1) and anti-VEGF (Figure 4, column 2). We only observed up-regulation of vWF and VEGF in the LT diabetics (Figure 4: A1, A2, D1, D2 and G1, G2). The retina showed up-regulation of vWF in vessels localised in the fibre layer (FL) and in the outer plexiform layer (OPL) (Figure 4: A1), while VEGF up-regulation was found in the OPL (Figure 4: A2). In this layer, co-localisation of vWF and VEGF was observed (Figure 4: A3). A similar co-expression of the two proteins (vWF and VEGF) was found in vessels along the iris stroma (Figure 4: D1, D2 and D3). In the cornea, the immunoreactivity for vWF and VEGF was observed in stromal vessels (Figure 4: G1, G2 and G3). Neither the ST diabetic group nor the controls exhibited immunoreactivity for vWF and VEGF in the retina, iris or cornea (Figure 4: rows H and I).

**Figure 5:**
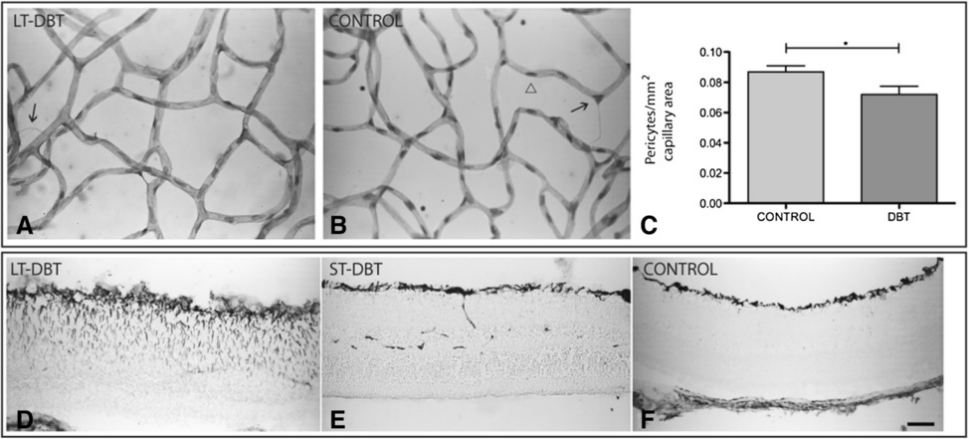
**Additional studies.** Tripsin digestive technique of the retina **(A, B). A**, LT-DBT capillary-net, arrows point to acellular capilary. **B**: vascular capillary-net from a retina between middle size vessels. Arrow points to pericytes and arrowhead to endothelial cells. **C**, Number of pericytes per capillary area was determined as indicated in methods section. Results are mean ± SEM of five rats in each group. *P <0.05. **D**, **E** and **F** are transverse retinal sections with GFAP immunostaining from LT-DBT rats **(D)**, ST-DBT rats **(E)** and controls **(F)**. Diabetic retinas **(D and E)** overexpressed GFAP in the fibre layer and in cells with the morphology and spatial organisation of Müller cells. Bar 50 μm.

The number of pericytes measured by the trypsin digestive technique was found to be significantly lower in the LT-diabetic group than in the controls (Figure 5)(p = 0.0298 unpair t-test p < 0.05; Mean ± SEM of Control 0.08670 ± 0.003975 N = 31; Mean ± SEM of DBT group 0.07206 ± 0.005373 N = 21). Positive immunoreactivity for GFAP was found in the FL and in cells morphologically similar to Müller cells. Staining was much more extensive in the LT diabetics than in the ST diabetics and controls (Figure 5).

## Discussion

We carried out an experimental study of diabetic retinopathy in an animal model of female type 2 diabetic rats fed on an HFD. The animals developed retinal and corneal neovascularization, as well as rubeosis iridis, after 110 weeks (two years) of diabetes. To our knowledge, this is the first report on ocular neovascularization in type 2 diabetic rats fed on an HFD.

Animal models are extremely important for the advancement of science and from time to time researchers report new models of diseases. However, the complexity of some diseases makes this task difficult, particularly when both systemic and local factors contribute to the illness, as in the case of diabetic retinopathy. Another element to consider is the cost of animal models, which should be reasonable. Big animals are expensive and difficult for researchers to afford [4]. So, these aspects convinced us to opt for the rat model of type 2 diabetes fed on an HFD. This model was previously characterised by other investigators [16,20] and recommended for studies on diabetic complications associated with obesity [21].

It should be mentioned that we did not find proliferative retinopathy changes in a previous study carried out in male type 2 diabetic rats of 90 weeks of disease, who were also fed on an HFD (unpublished data). Although the duration of diabetes is five months longer in this survey of female rats, other factors can lead to the growth of proliferative changes in these animals. The potential influence of oestrogens in the progression of diabetes in rats is known [22] and this is possibly enhanced by depletion of SHBG in female rats. It has been reported that female rats are less sensitive to insulin than males of all age groups and also more susceptible to the rapid development of a severe form of diabetes. This is still controversial in the scientific literature. In any case, the existence of sex receptors in the rat eye may play a role in retinopathy development. These receptors are also found in the human eye [23,24]. The chemical compound of the HFD used in our research studies in male and female diabetic rats was the same and we know that this type of diet increases the risk of cardiovascular disease and promotes cell damage [25-27]. So, an early onset of diabetes (STZ injection at day two, followed by HFD at eight weeks onwards), a longer duration, known clinical risk factors and female sex might have been key elements in the development of ocular neovascularization.

The first retinal morphological changes observed in diabetic rats described in the scientific literature included loss of pericytes and the presence of acellular and collapsed capillaries. These changes lead to retinal ischaemia [28]. We found a significantly lower number of pericytes in LT diabetic rats than in the controls, which is in accordance with previous studies.

In our survey, the morphology of pre-retinal neovascularization was consistent with that reported by other authors [3,29,30]. Intra-retinal neovessels were seen in the inner part of the retina (Figure 2). This type of vessel always shows positive immunoreactivity of the vWF Factor and VEGF as well as co-localisation of them. It is known that vWF is a marker of endothelial dysfunction and widely used to study angiogenesis in cancer and ocular diseases [31,32]. Staining of this marker was not seen in the vessels of the ST diabetics and controls. It should be noted that all LT diabetic animals had pre-retinal and/or intra-retinal neovessels. Similarly, all LT diabetics showed a thick iris, as well as rubeosis iridis, a known consequence of severe retinal hypoxia and the release of angiogenic factors that lead to neovascular glaucoma in humans. This, in turn, can cause corneal endothelial dysfunction with bullous keratopathy, corneal edema and epithelial erosions, release of inflammatory cytokines, limbal stem cells deficiency and corneal neovascularization, which was clinically observed in three of the LT diabetic animals in our study.

## Conclusions

An animal model mimics a human disease and it can be considered as a platform for the study of mechanisms and therapeutic agents. For this reason, several models have been developed in ophthalmology, such as the oxygen-induced retinopathy model [33] and the proliferative diabetic retinopathy model using gene therapy [30]. In our model, the streptozotocin injection, HFD, earlier onset and longer duration of diabetes and probably female sex are considered major contributing factors to disease growth. Future investigations will confirm the usefulness of this diabetic retinopathy animal model.

## Abbreviations

HFD: High-fat diet; STZ: Streptozotocin; GFAP: Glial-fibrillary-acidic-protein; VEGF: The vascular endothelial growth factor; vWF: Von Willebrand factor; SHBG: Sex hormone binding globulin; ISO: International organization for standardization; H&E: Haematoxylin and eosin; PAS: Periodic acid-schiff stain; SD: Standard deviations; LT-DBT: Long-term diabetic rats; ST-DBT: Short-term diabetic rats; FL: Fibre layer; OPL: Outer plexiform layer.

## Competing interests

The authors declare that they have no competing interests.

## Authors’ contributions

JEM: AB, MT, ES, FG; JOC: MT, FG; JEG: MT, ES, FG. All authors read and approved the final manuscript.
